# Supplementary material for: Effects of Different Phospholipid Sources on Growth and Gill Health in Atlantic Salmon in Freshwater Pre-Transfer Phase
Source: Animals (Basel). 2023 Feb 24;13(5):835. doi: 10.3390/ani13050835 (PMC10000100; doi:10.3390/ani13050835)
Supplement: Supplementary file 1 [file animals-13-00835-s001.zip › animals-2213812-supplementary.pdf]

Supplementary Table S1. Fish weight, fish weight gain, feed intake and FCR for the 53d of trial diet feeding in freshwater pre-transfer period

Diet	Initial weight (g/fish)	Final weight (g/fish)	Weight gain (g/fish)	Feed Intake (g/fish)	FCR
CONTROL	75± 5	159± 7	84± 4	108± 23	1.28±0.23
KM4	72± 4	156± 10	84± 7	112± 14	1.34± 0.25
KM8	73± 3	162± 12	89± 10	106± 8	1.18± 0.07
KM12	74± 5	164± 9	89± 8	111± 17	1.23± 0.09
MARPL	74± 4	155± 10	81± 7	96± 9	1.18± 0.17
VEGPL	73± 4	150± 13	77± 9	102± 22	1.33± 0.26

Data given as average ± standard deviation for 4 replicate tanks from each diet with initial weight at start of freshwater pre-transfer trial diet feeding, final weight at end of freshwater pretransfer phase and weight gain based on the average weight of all fish in the tank. High survival in the pre-transfer freshwater feeding period with 5 mortalities in total out of the 960 total fish (40 fish per tank in 24 tanks).

Supplementary Table S2. Fish weight and fish weight gain for common seawater tank post-transfer period with fish fed the same commercial diet over 98d.

Diet	Initial weight (g/fish)	Final weight (g/fish)	Weight gain (g/fish)
CONTROL	163± 7	790± 29	627± 29
KM4	162± 19	791± 72	629± 65
KM8	177± 28	785± 57	609± 29
KM12	164± 12	793± 16	629± 28
MARPL	164± 5	791± 41	626± 37
VEGPL	159± 18	765± 32	606± 19

Data given as average ± standard deviation for 4 replicate tanks from each diet fed during pre-transfer freshwater period with initial weight at start of seawater phase, final weight at end of seawater phase and weight gain based on the average weight of all fish in the tank. High survival in the seawater phase with 3 mortalities and 2 missing fish at final sampling in total out of the 330 total fish (not including fish removed at intermediate time points) that were in the common seawater tank through the whole seawater phase.
